# Why do vertical glabellar lines form? A guideline on botulinum neurotoxin injection

**DOI:** 10.1111/jocd.16534

**Published:** 2024-08-12

**Authors:** Jovian Wan, Kyu‐Ho Yi

**Affiliations:** ^1^ Asia‐Pacific Aesthetic Academy Hong Kong Hong Kong; ^2^ Division in Anatomy and Developmental Biology, Department of Oral Biology Human Identification Research Institute, BK21 FOUR Project, Yonsei University College of Dentistry Seoul South Korea; ^3^ Maylin Clinic (Apgujeong) Seoul South Korea

**Keywords:** aging, botulinum toxins, cosmetic techniques, facial muscles

## 1 INTRODUCTION

Botulinum neurotoxin (BoNT) injections directed at the procerus muscle are frequently employed by aesthetic practitioners to induce chemical denervation, leading to prolonged yet reversible muscle relaxation. This approach aims to improve the appearance of glabellar lines or rhytids and has been extensively documented in the literature.^1^, ^2^, ^3^, ^4^, ^5^

**FIGURE 1:**
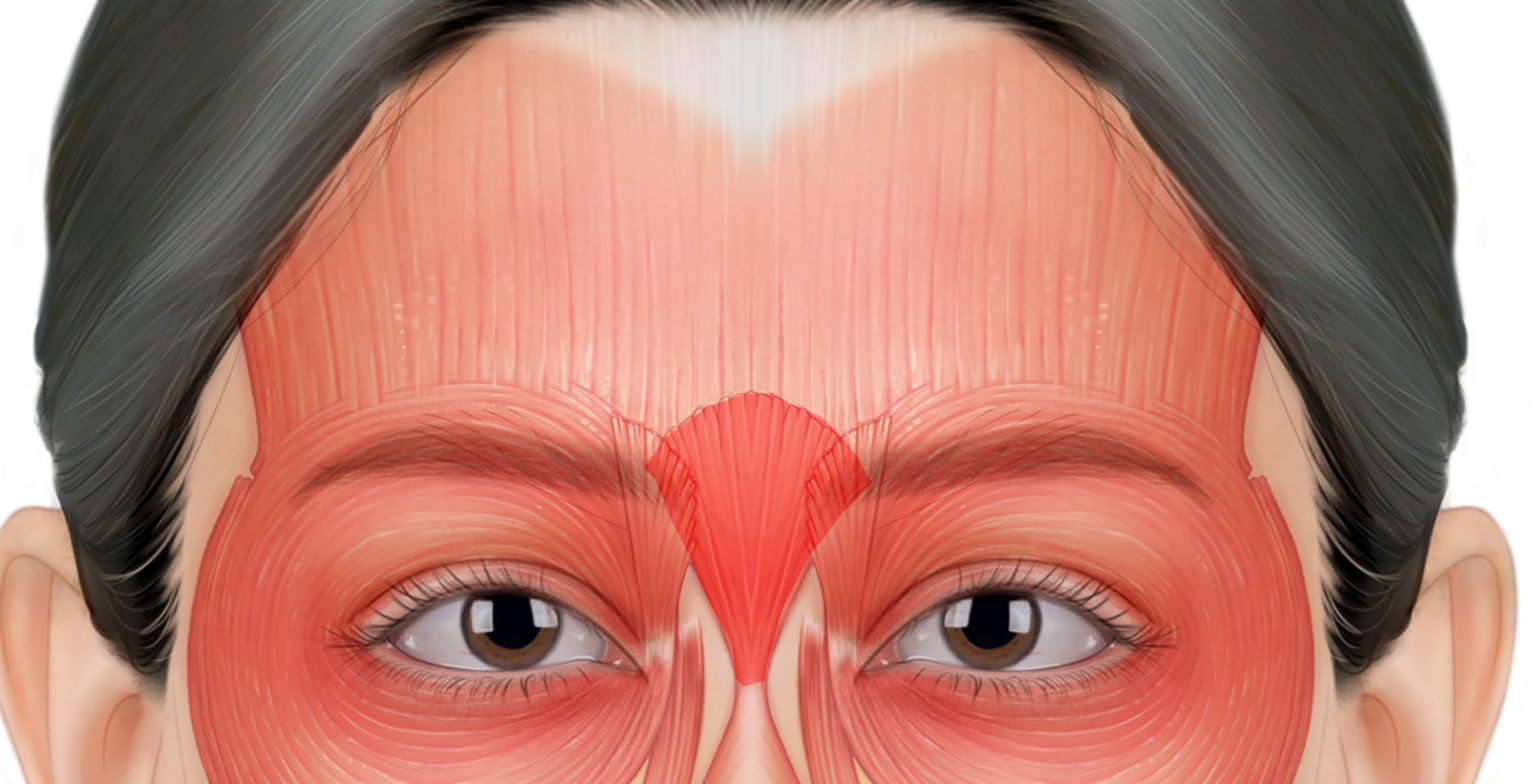
The procerus muscle, indicated by the red shaded.

The procerus is a small pyramidal‐shaped muscle located in the glabella region (Figure 1). It originates from the fascia near the junction of the nasal bones and the upper lateral nasal cartilage.^6^ Previous anatomical studies on cadavers have revealed that it consists of superficial and deep layers and extends to the frontalis muscle superiorly and the nasalis muscle inferiorly.^7^ Cho et al.^1^ performed an ultrasound‐guided investigation of the anatomy of the procerus in Asian populations, aiming to measure its depth and thickness. The authors identified two distinct morphological types of the procerus based on the analysis of the ultrasound images obtained.

**FIGURE 2:**
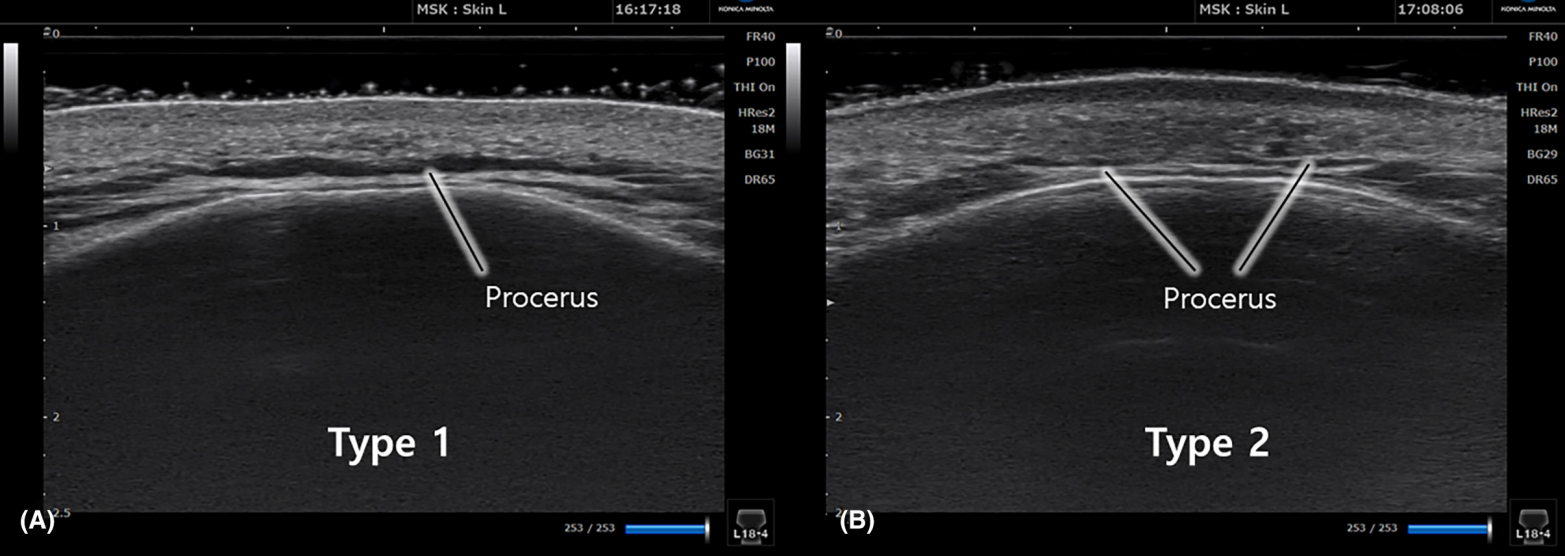
There are two distinct types of procerus muscles based on their morphology in ultrasonographic imaging when viewed in the transverse plane. (A) depicts type I procerus muscle, (B) represents type II procerus muscle.

Type I, seen in 79.7% of cases (55 of 69), had a clearly observable procerus muscle distinguishable from adjacent muscles in transverse‐view US images. Type II, observed in 20.3% of cases (14 of 69), did not show the procerus at the midline of the glabella, instead presenting as two lateral portions with a thin band‐like structure at the midline (Figure 2). The width of the procerus could not be measured in some Type I and Type II cases due to limitations in the US view. The thickness of the procerus at the sellion was measurable in all cases, with Type I muscles being significantly thicker than Type II (1.6 ± 0.6 mm vs. 1.3 ± 0.4 mm, *p* = 0.044). Conversely, in type II, the procerus muscle was not visualized along the midline of the glabella (Figure 2).

This classification of the procerus morphology has proven to be a valuable asset in our clinical practice and our aim is to integrate insights from existing literature to develop an improved injection protocol for the safe and effective administration of BoNT in addressing glabellar lines associated with type II procerus morphology. This protocol is established based on an innovative understanding of type II procerus contraction patterns.

## 2 INNOVATIVE IDEAS

**FIGURE 3:**
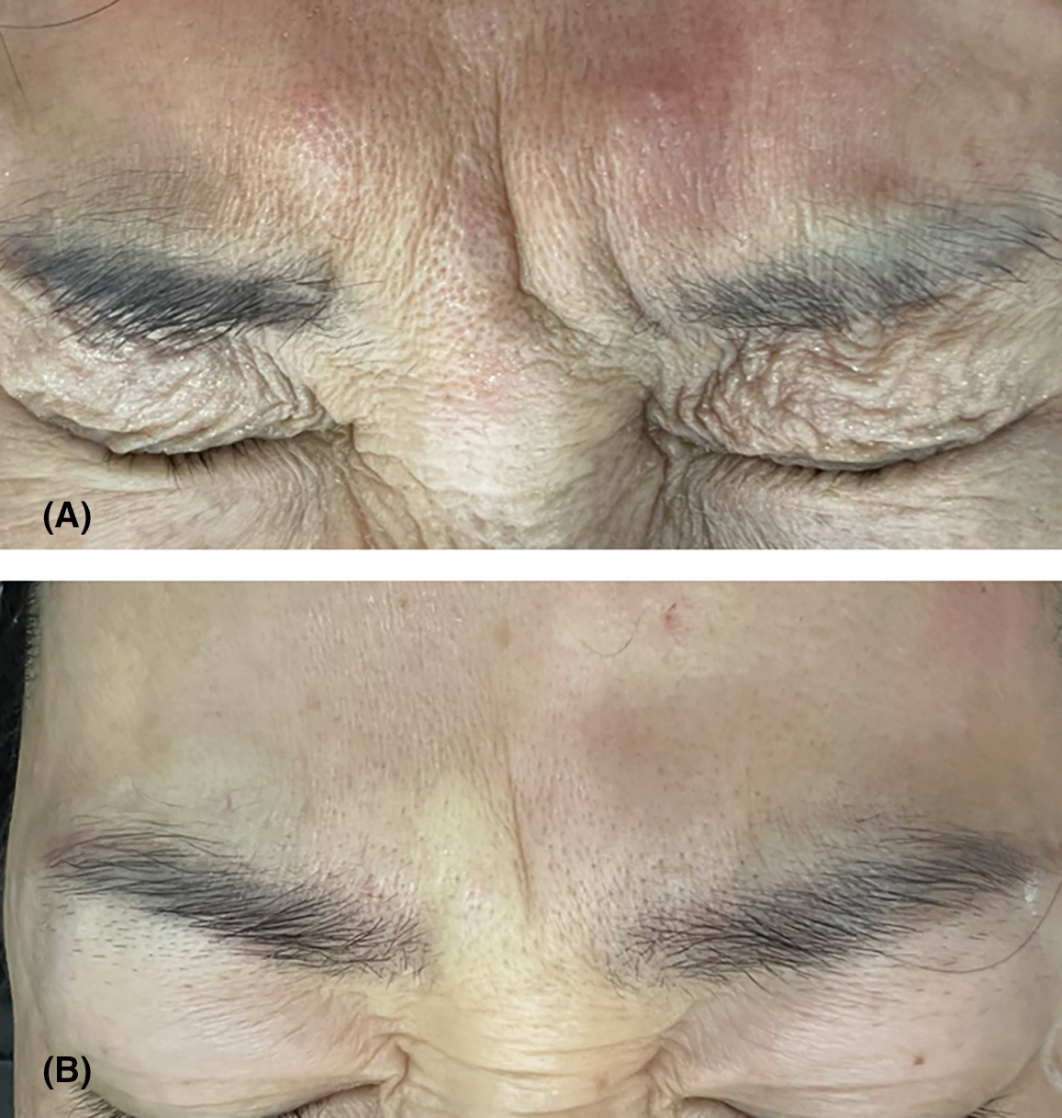
Panel (A, B) shows frowning pattern shows vertical wrinkles due to obsence of muscle belly at the center. We term this the “glabellar vertical line,” featuring a single midline wrinkle, suggesting procerus muscle absence in the midline of the glabella.

According to Cho et al.,^1^ the frowning pattern of the glabella can be classified into two types based on ultrasound imaging, which correlates with the morphology of the procerus. Type A represents the typical glabellar frowning pattern, characterized by vertical wrinkles formed by the contraction of the corrugator supercilii and procerus muscles. In contrast, Type B glabellar frowning pattern exhibits an upward elevation of vertical wrinkles caused by excessive contraction of the frontalis muscle during frowning. We are particularly interested in exploring the Type B glabellar frowning pattern, which we will refer to as the “glabellar vertical line” pattern. As illustrated in Figure 3, this pattern is characterized by a single vertical line in the midline of the glabella, indicating the absence of the procerus muscle belly in the midline of the glabella.

**FIGURE 4:**
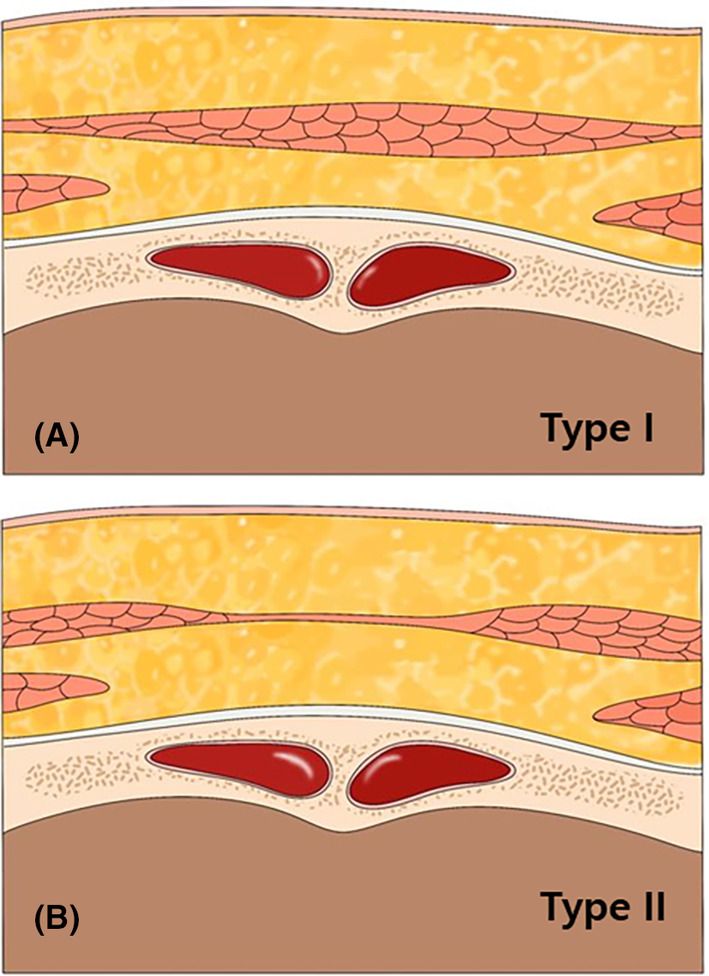
Illustration of the type II procerus muscle, characterized by a relatively weaker muscle belly in the middle (black arrows) compared to the more consistent muscle belly of Type I (black triangular arrow) (A). The type II procerus muscle is implicated in the formation of the vertical glabellar line (B).

In a retrospective observational case study conducted by authors at a private clinic, 12 Korean patients were randomly selected, consisting of three males and nine females, who had not received any aesthetic treatments in the previous 6 months. Prior to the study, informed consent was obtained from all participants. The age of the participants ranged from 33 to 48 years old. Patients with a history of botulinum neurotoxin, fillers, chemical peels, and microdermabrasion were excluded from the study. The study analyzed the classification pattern of the glabellar frowning observed in the patients. Among the 12 patients, 11 exhibited a vertical glabellar line frowning pattern associated with type II procerus muscle, as observed through ultrasound. These patients received botulinum toxin injections targeting the procerus muscle, which effectively diminished the line. This suggests that the type II procerus muscle, with a relatively weaker muscle belly in the middle compared to the more consistent muscle belly of type I, is implicated in the formation of the vertical glabellar line (Figure 4).

**FIGURE 5:**
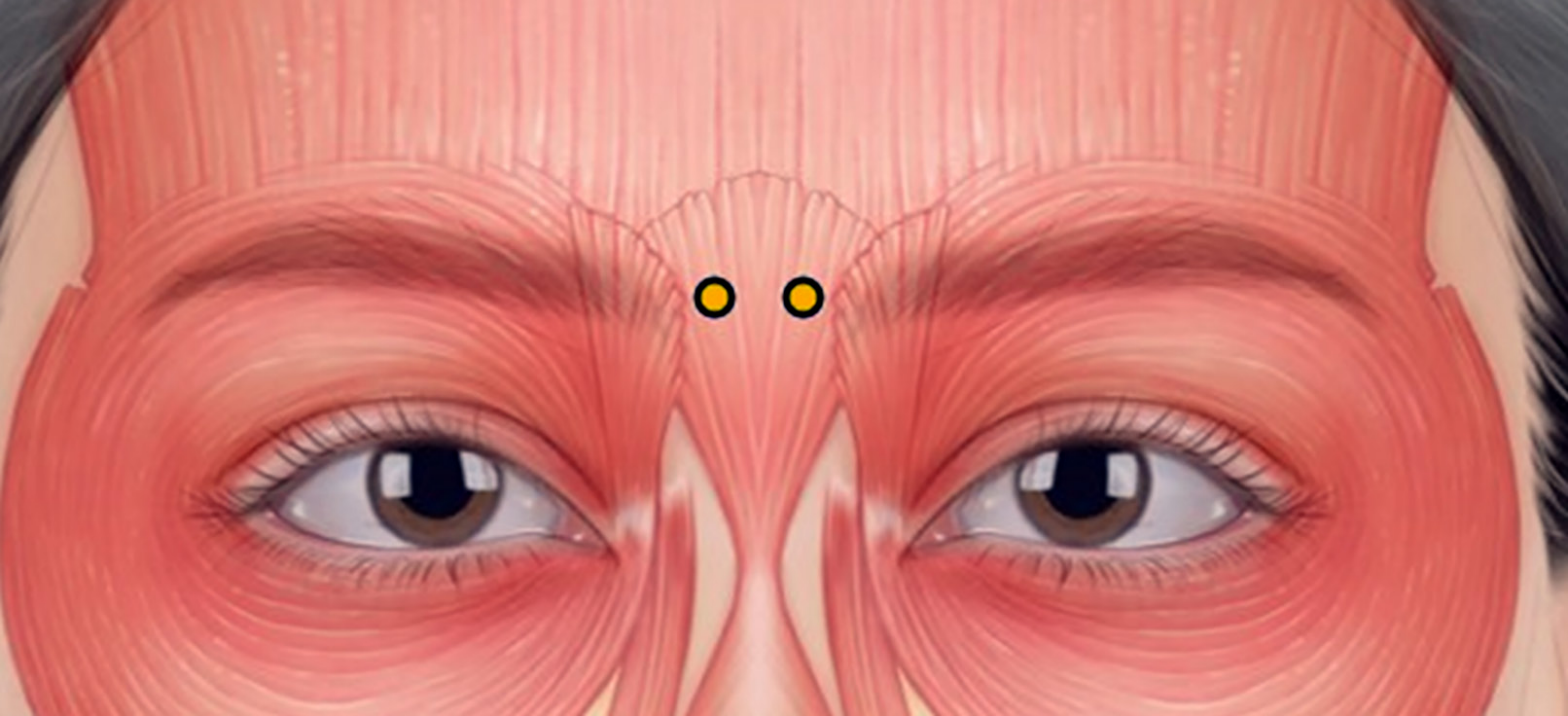
The recommended injection points for treating the glabellar vertical line (type II procerus). Instead of targeting the centre of the procerus where no muscle belly exists, injections should be precisely administered at two points (yellow dots) slightly lateral to the midline of the glabella. The authors recommend administering 2 units of botulinum neurotoxin at each injection point, although dosage may vary based on individual patient needs.

When addressing the glabellar vertical line, it is crucial to avoid directing BoNT injections towards the centre of the procerus, where the muscle belly is absent. Rather, injections should be precisely aimed at two points slightly lateral to the centre of the glabella's midline, targeting the remaining sections of the procerus muscle, as indicated in Figure 5. This targeted approach ensures that BoNT is administered effectively to the muscles responsible for the formation of the glabellar vertical line, leading to optimal treatment outcomes. The authors advise administering 2 units of BoNT at each injection point, although dosage may vary depending on the patient's specific requirements.

**FIGURE 6:**
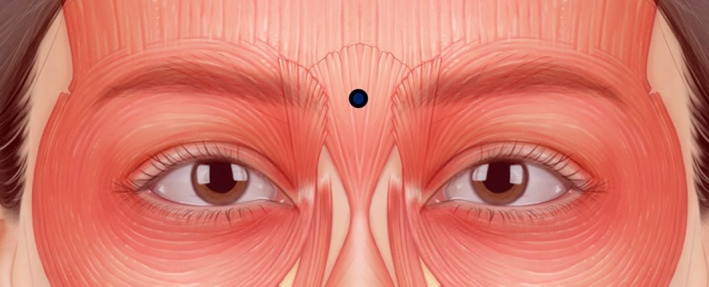
The recommended injection points for treating the typical glabellar frowning pattern (type I procerus). Injections should be administered at the centre (blue dot) of the procerus muscle, as depicted. The authors suggest administering 4 units of botulinum neurotoxin at this injection point, with dosage adjustments tailored to individual patient needs.

As discussed, the glabellar frowning pattern observed in type I procerus muscle typically manifests as vertical wrinkles formed by the contraction of both the corrugator supercilii and procerus muscles. When addressing this pattern, injections should be directed at the centre of the procerus muscle, as indicated in Figure 6. The authors recommend administering 4 units of BoNT at this injection point, adjusted based on individual patient needs for optimal treatment outcomes.

## 3 DISCUSSION

The glabellar vertical line frowning pattern associated with type II procerus morphology is a novel observation not yet described in the literature. While previous research extensively explored variations in procerus muscle anatomy and function concerning glabellar frowning patterns, the specific correlation between type II procerus morphology and the appearance of a single vertical line along the midline of the glabella remains unaddressed.^1^, ^7^, ^8^, ^9^, ^10^ This unique pattern, marked by the absence of the procerus muscle belly in the midline of the glabella, underscores the complexity of facial muscle dynamics. Further investigation into its clinical significance is warranted. Based on the evidence of the anatomical shape of the procerus, specifically the bilateralized structure observed in Type II procerus, it would be appropriate to target the muscle belly with a minimal dosage of botulinum toxin. This targeted approach ensures precise delivery of the toxin to the muscle, potentially improving the efficacy of the treatment. While the standard single injection technique may diffuse enough to treat both types of procerus, the proposed dual injection technique is suggested to ensure adequate treatment of the bilateralized procerus muscle, particularly in Type II cases. However, there is a limitation of the study that needs further clinical data comparing the efficacy of single versus dual injection techniques in treating both types of procerus would be beneficial to substantiate this approach. One another limitation of our study is the lack of research on the presence of the described anatomical patterns of the procerus muscle across different ethnicities. Our findings are based on a specific patient population, and we did not investigate whether these morphological variations are consistent among diverse ethnic groups. Future studies should include a broader range of ethnicities to determine the generalizability of our results.

## 4 CONCLUSION

In conclusion, our identification of the glabellar vertical line frowning pattern associated with type II procerus morphology represents a novel observation not previously described. This finding underscores the complexity of facial muscle dynamics and warrants further investigation into its clinical significance to refine treatment approaches for optimal outcomes.

## AUTHOR CONTRIBUTIONS

**Kyu‐Ho Yi and Jovian Wan**: Conceptualization. **Jovian Wan** and **Kyu‐Ho Yi**: Writing—original draft preparation. **Jovian Wan and Kyu‐Ho Yi**: Writing—review and editing. **Kyu‐Ho Yi** and **Jovian Wan**: Visualization. **Kyu‐Ho Yi**: Supervision. All authors have reviewed and approved the article for submission.

## FUNDING INFORMATION

The authors received no financial support for the research, authorship, and publication of this article. The products utilized in this study were donated by the injectors for the purposes of this study.

## CONFLICT OF INTEREST STATEMENT

The authors declared no potential conflicts of interest with respect to the research, authorship, and publication of this article. This study was conducted in compliance with the principles set forth in the Declaration of Helsinki.

## ETHICS STATEMENT

This study was performed in line with the principles of the Declaration of Helsinki.

## Data Availability

The data that support the findings of this study are available from the corresponding author upon reasonable request.
